# Efficient isolation and proteomic analysis of cell plasma membrane proteins in gastric cancer reveal a novel differentiation and progression related cell surface marker, R-cadherin

**DOI:** 10.1007/s13277-016-5032-z

**Published:** 2016-03-30

**Authors:** Bo Chen, Qi-Cong Luo, Jian-Bo Chen, Li-E Lin, Ming-Xu Luo, Hong-Yue Ren, Pei-Qiong Chen, Lian-Guo Shi

**Affiliations:** 1 Department of Gastrointestinal Surgery, Xiamen Cancer Hospital of The First Affiliated Hospital of Xiamen University, 361003 Xiamen, Fujian China; 2 Central Laboratory, Xiamen Cancer Hospital of The First Affiliated Hospital of Xiamen University, 361003 Xiamen, Fujian China; 3 Department of Oncology, Chenggong Hospital affiliated to Xiamen University, 361003 Xiamen, Fujian China; 4 Department of Radiation Oncology, Xiamen Cancer Hospital of The First Affiliated Hospital of Xiamen University, 361003 Xiamen, Fujian China; 5 Department of Biobank, Xiamen Cancer Hospital of The First Affiliated Hospital of Xiamen University, 361003 Xiamen, Fujian China; 6 Department of Pathology, Xiamen Cancer Hospital of The First Affiliated Hospital of Xiamen University, 361003 Xiamen, Fujian China

**Keywords:** Gastric adenocarcinoma, Cell surface proteins, R-cadherin, Tumor differentiation, Tumor progression, Tumor biomarker

## Abstract

Cell plasma membrane proteins, playing a crucial role in cell malignant transformation and development, were the main targets of tumor detection and therapy. In this study, CyDye/biotin double-labeling proteomic approach was adopted to profile the membrane proteome of gastric cancer cell line BGC-823 and paired immortalized gastric epithelial cell GES-1. Real-time PCR, Western blotting, and immunohistochemical staining were used to validate the differential expression of a novel identified cell surface marker R-cadherin in gastric cancer cells and tissues. Clinicopathological study and survival analysis were performed to estimate its roles in tumor progression and outcome prediction. Real-time PCR and Western blotting showed that the expression level of R-cadherin in gastric cancer were significantly lower than non-cancerous epithelial cell and tissues. Clinicopathological study indicated that R-cadherin was dominantly expressed on cell surface of normal gastric epithelium, and its expression deletion in gastric cancer tissues was associated with tumor site, differentiation, lymph node metastasis, and pTNM (chi-square test, *P* < 0.05). Those patients with R-cadherin positive expression displayed better overall survivals than negative expression group (log-rank test, *P* = 0.000). Cox multivariate survival analysis revealed lacking the expression of R-cadherin was a main independent predictor for poor clinical outcome in gastric cancer (RR = 5.680, 95 % CI 2.250–14.341, *P* < 0.01). We have established a fundamental membrane proteome database for gastric cancer and identified R-cadherin as a tumor differentiation and progression-related cell surface marker of gastric cancer. Lacking the expression of R-cadherin indicates poor prognosis in patients with gastric cancer.

## Introduction

Gastric cancer (GC) is the second most common cause of cancer death worldwide, although its incidence and mortality have fallen dramatically over the last 50 years [1, 2]. According to the report of Global Cancer Statistics, nearly three quarters of new cases of GC were diagnosed in Asia and nearly half of these cases occurred in China [3, 4]. Surgery remains the primary curative treatment for GC [5, 6]; however, successful early detection and radical resection of GC are hampered by lacking of highly sensitive and specific biomarkers [7]. Therefore, screening more specific and sensitive early diagnostic biomarkers is an effective way to improve overall outcomes of GC [8, 9].

Despite significant improvements in systemic chemotherapy over the last two decades, the prognosis of advanced GC remains frustrating. As a part of comprehensive treatment for gastric carcinoma, targeted chemotherapy and its related treatment have wide development in recent years [10, 11]. Although progress has been made, thanks to trastuzumab in partial HER2 positive GC, antiangiogenic drugs have produced conflicting results and EGFR-inhibitors have failed to show major improvements [12, 13]. Discovering new therapeutic targets is a valuable and challenging work to improve the prognosis of GC.

Circulating tumor cells (CTCs), acting as important contributor and indicator for cancer metastasis [14], have been studied as promising prognostic and predictive tumor-derived biomarkers in the bloodstream of patients with gastrointestinal malignancies [15–17]. Both the basic magnetic bead separation and the latest micro-fluidic chips applied for CTC enrichment and isolation all depend on the immunological recognition of the cell surface markers [18, 19]. Due to lacking of tumor-specific cell surface markers, most of the current CTC detection methods were obliged to base on the epithelial markers such as EpCAM. Most of these epithelial markers might be “blind” to the most dangerous cancer cells present in the circulation, which may be going through epithelial-mesenchymal transition (EMT) and losing the expression of epithelial markers [20, 21]. Therefore, exploring tumor-specific cell surface marker is essential to improve the isolation efficiency of CTCs from tumor patients.

Membrane proteins expressed on cell surface, which play crucial roles in the whole process of cancer development, were the major targets of the majority of antitumor reagents [22, 23]. A comprehensive membrane proteomic study of GC will not only benefit for further understanding the mechanisms of tumorigenesis of GC but also help to discover some novel diagnostic and therapeutic targets [24].

As a kind of hydrophobic protein, extraction and enrichment of cell surface proteins from tumor cells were extremely difficult and the membrane proteomic database in GC is still rare [25]. In order to explore the composition and characteristics of cell surface proteins in GC, we adopted a novel double-labeling membrane proteomic strategy to profile the cell surface proteins in GC as described before [26]. In our study, a series of membrane proteins including a differential expressed protein R-cadherin were identified from GC cell line BGC-823. In addition, we investigated the relationship between R-cadherin expression and the clinicopathological characteristics of GC. Its prognostic value was also estimated in this study.

## Materials and methods

### Clinical samples

Twenty-five pairs of fresh frozen gastric adenocarcinoma and the adjacent non-cancerous mucosa tissues were taken from tumor Biobank of The First Affiliated Hospital of Xiamen University. Tissue microarray of human GC (OD-CT-DgStm01-007 and HStm-Ade178Sur-01) was obtained from National SOBC Biobank, which included 169 GC specimens, 80 samples of adjacent normal mucosa to gastric carcinoma. Each patient had signed informed consent for this research. This study protocol was approved by the ethical committee of The First Affiliated Hospital of Xiamen University.

Among these 169 GC samples, 117 of these patients were men (69.2 %) and 52 (30.8 %) were women. The mean age of the cohort was 59.5 ± 11.2 years (median 60 years, range 28–84 years). The levels of differentiation were used to classify grading as the following: well and moderate (*n* = 37, 21.9 %) and poor and undifferentiated (*n* = 132, 78.1 %). Tumor staging was assessed using the seventh edition of the tumor, node, metastasis (TNM) system according to the Union for International Cancer Control (UICC) and the American Joint Committee on Cancer (AJCC). They were classified as IA (*n* = 10, 5.9 %), IB (*n* = 14, 8.3 %), IIA (*n* = 14, 8.3 %), IIB (*n* = 20, 11.8 %), IIIA (*n* = 35, 20.7 %), IIIB (*n* = 49, 29 %), IIIC (*n* = 9, 5.3 %), and IV (*n* = 18, 10.7 %). One hundred twenty-three cases were followed-up for at least 5 years.

### Cell surface labeling and membrane protein separation

Human GC cell lines BGC-823, MGC-803, and SGC-7901, were purchased from the Type Culture Collection of the Chinese Academy of Sciences (Shanghai, China). The immortalized human gastric epithelial cell line GES-1 was obtained from ATCC, USA. Cells were maintained in RPMI-1640 supplemented with 10 % fetal bovine serum (FBS), 100 U/ml penicillin, and 100 μg/ml streptomycin in a humidified incubator with 5 % CO_2_ at 37 °C. All culture materials were purchased from GIBCO, USA. Cell Surface Protein Isolation Kit (89,881) is a product of Pierce, USA. CyDye™ DIGE fluors dyes are all products of GE Healthcare, USA.

BGC-823 and GES-1 were cultured in 100-mm dishes until reaching 70–80 % (approximately 6 × 10^6^ cells/dish). Cells were quickly washed thrice with ice-cold Hanks’ balanced salt solution (HBSS) and then cultured in fresh serum-free RPMI-1640 media for 16 h. After that, cells were washed thrice with HBSS again and then incubated in the CyDye/Cy3 working liquid (1 × 10^7^ cells/nmol) for 15 min at 4 °C. After washed by HBSS thrice to remove the unlabeled Cy3, 10-ml sulfo-NHS-SS-biotin working solutions which provided by Cell Surface Protein Isolation Kit was added to the Cy3-tagged cells and rocked gently for 30 min at 4 °C to ensure the efficient of labeling reaction. In order to stop labeling reactions, 1 ml of L-lysine solution (10 mmol) and 10-ml quenching solution (PBS with 100 mmol L-glycine) was added to each culture cell. The double-tagged cells were scraped from dishes gently and transferred into 50-ml centrifuge tube to collect cell pellets (500*g* for 3 min at 4 °C). Cell pellets were washed thrice with 5 ml of TBS gently and dissolved in 500-μl lysis buffer (7 M urea, 2 M thiourea, 2 % CHAPS, 2 % Triton-X, 2 % ampholyte, 1%DTT). Next, the cell lysates were centrifuged at 10,000*g* for 2 min at 4 °C, then the supernatant was incubated with 500 μl of slurry of avidin beads in a column (prewashed thrice with wash buffer) for 60 min at room temperature on a rocking platform. The column was centrifuged for 1 min at 1000*g*, and the flow-through was collected. Then the beads were washed thrice using 500 μl of wash buffer with 1 % protease inhibitors. Finally, to elute the biotinylated proteins from the beads, 500 μl of sodium dodecyl sulfate-polyacrylamide gel electrophoresis (SDS-PAGE) sample buffer (120 mmol/l Tris-HCl pH 6.8, 20 % glycerin, 4 % SDS, 3 % β-mercaptoethanol, 1 % protease inhibitors) was added to the column and shook up and down for 60 min at room temperature. Then the protein samples were collected following centrifugation for 1 min at 1000*g*. The extracted proteins were quantified with Bradford’s reagent (Bio-Rad laboratories, USA). Each 30-μg extracted membrane protein of BGC-823 and GES-1 was subjected to SDS-PAGE gel (12.5 % separation gel and 5 % spacer gel) in three lanes, followed by protein electrophoresis in GE MiniVE (80 V for 3 h). The gel was fluorescent imaged and analyzed with Typhoon imager 9410 (GE Healthcare) using the 488-nm laser. After the fluorescence imaging, the gel was post-stained by improved silver staining. The consistent protein strips presented in both fluorescence and silver dyeing were selected and matched and cut off from gel for subsequent protein digestion and liquid chromatography-tandem mass spectrometry (LC-MS/MS).

### Protein digestion and LC-MS/MS

The silver-stained protein bands were detained with 30 % ACN/100 mmol ammonium bicarbonate and dried in a vacuum centrifuge. The in-gel proteins were reacted with dithiothreitol (10 mmol DTT/100 mmol ammonium bicarbonate) for 30 min at 56 °C, then alkylated with iodoacetamide (200 mmol indoleacetic acid/100 mmol ammonium bicarbonate) in the dark at room temperature for 30 min. Gel bands were briefly rinsed with 100 mmol ammonium bicarbonate and ACN, respectively. After that, gel bands were digested overnight in 12.5 ng/μl trypsin in 25 mmol ammonium bicarbonate. The peptides were extracted three times with 60 % ACN/0.1 % trifluoroacetic acid (TFA). The extracts were pooled and dried completely by a vacuum centrifuge. Ettan™ MDLC system (GE Healthcare, USA) was applied for desalting and separation of tryptic peptides mixtures. In this system, samples were desalted on RP trap columns (Zorbax 300 SB C18, Agilent Technologies, USA) and then separated on a RP column (150 μm i.d., 100 mm length, Column Technology Inc., Fremont, CA). Mobile phase A (0.1 % formic acid in HPLC-grade water) and the mobile phase B (0.1 % formic acid in acetonitrile) were selected. Twenty micrograms of tryptic peptide mixtures was loaded onto the columns, and separation was done at a flow rate of 2 μl/min by using a linear gradient of 4–50 % buffer B for 50 min, 50–100 % buffer B for 4 min, and 100 % buffer B for 6 min. LTQ Velos (Thermo Scientific, USA) equipped with a micro-spray interface was connected to the LC setup for eluted peptides detection. Data-dependent MS/MS spectra were obtained simultaneously. Each scan cycle consisted of one full scan mass spectrum (*m*/*z* 300–1800) followed by 20 MS/MS events of the most intense ions with the following dynamic exclusion settings: repeat count 2, repeat duration 30 s, and exclusion duration 90 s. MS/MS spectra were automatically searched against the ipi.HUMAN.v3.53 using the BioworksBrowser rev.3.1 (Thermo Electron, San Jose, CA). Protein identification results were extracted from SEQUEST out files with Build Summary. The peptides were constrained to be tryptic and up to two missed cleavages were allowed. Carbamidomethylation of cysteines was treated as a fixed modification, whereas oxidation of methionine residues was considered as variable modifications. The mass tolerance allowed for the precursor ions was 2.0 Da and fragment ions was 0.8 Da, respectively. The protein identification criteria were based on Delta CN (≥0.1) and cross-correlation scores (Xcorr, one charge ≥1.9, two charges ≥2.2, three charges ≥3.75).

### Real-time quantitative PCR analysis (qRT-PCR)

Total RNA was extracted from culture cells, and 17 pairs of fresh frozen gastric adenocarcinoma and the adjacent non-cancerous mucosa tissues using Trizol reagent (Invitrogen, USA) and then reverse transcripted to synthesize the first-strand cDNA using Qiagen OneStep RT-PCR Kit (Qiagen, USA) according to the instructions of the manufacturer. The transcription levels were detected by THUNDERBIRD SYBR qPCR Mix kit (TOYOBO, Japan) to monitor the amplification. β-actin was used as an endogenous control to normalize expression. PCR reactions in triplicate were performed by PCR and initial denaturation at 95 °C for 5 min followed by 45 cycles, each consisting of 10 s at 95 °C, 20 s at 56 °C, and 20 s at 72 °C. The △Ct method was adopted for analysis. First, the cycle number at the threshold level of fluorescence (Ct) for each sample was determined. Next, △Ct value was calculated.

Primer sequences used were as follows:

- h-actin-fCGAGCGGGAAATCGTGCGTGACATTAAGGAGA
- h-actin-rCGTCATACTCCTGCTTGCTGATCCACATCTGC
- h-CDH4-fCTATGACTCCCTGCTGGTCTTC
- h-CDH4-rAATCCTCTTCACCACCTCCATA

The 2−△△Ct method for relative quantification of gene expression was used to determine the messenger RNA (mRNA) expression levels.

### Western blotting

In vitro cultured GC cells BGC-823, MGC-803, and SGC-7901 and human gastric epithelial cell GES-1 were harvested and lysed with RIPA lysis buffer (Beyotime, China) for 15 min on ice. One hundred milligrams tissue homogenate of each frozen samples which came from eight pairs of fresh frozen gastric adenocarcinoma and the adjacent non-cancerous mucosa tissues were lyzed with RIPA lysis buffer for 30 min on ice. After centrifugation at 13,000*g* for 10 min, the concentration of proteins was measured using Bradford’s reagent (Bio-Rad laboratories, USA). The protein samples were denatured by boiling for 5 min and load onto 12.5 % SDS-PAGE gel for electrophoresis. The proteins were transferred onto PVDF membrane (Millipore, USA) and incubated in blocked solution (5 % nonfat milk in PBS) for 1 h at room temperature. The anti-R-cadherin antibody (sc-6622, Santa Cruz, USA) was added into blocking solution (1:1000) and incubated at 4 °C for 16 h, followed by washing and incubated again with horseradish peroxidase-conjugated goat anti-rabbit secondary antibodies (1:5000 dilution) for 1 h at room temperature. Protein expression was normalized against β-actin expression (Sigma, USA). Membranes were washed three times for 5 min each; then, ECL kit (GE Healthcare, USA) was applied for blot imaging according to the instructions of the manufacturer.

### Immunohistochemistry

Immunohistochemistry for R-cadherin was performed on tissue microarray with Maxvison™ one step immunohistochemistry kit (Maixin Biotech, China). Briefly, tissue microarray was dewaxed in xylene and brought to water through graded alcohols. The antigens were repaired by high-pressure cooking with citric acid repair solution (Maixin Biotech, China, 1:100) and incubated with anti-R-cadherin antibodies (1:100 dilution) at 4 °C overnight. After the antigen immunoreactivity, tissue microarray was washed thrice with PBS and incubated with Maxvison™ for 15 min at room temperature and rinsed thrice with PBS. The resultant immune peroxidase activity was developed in DAB Detection Kit (Maixin Biotech, China) for 3 min and counterstained with Harris’ hematoxylin. Appropriate negative controls were performed by omitting the primary antibody. As positive controls, paraffin-embedded liver sections with known immunoreactivity for R-cadherin were used. The percentages of positively stained cells were obtained by counting at least 1000 cells in each case by two independent experienced pathologists (S.T. and S.V.) blinded to the clinical data with complete observer’s agreement. Evaluation of immunostaining was analyzed according to both the percentage of positive-staining cells and the intensity of membrane staining. Intensity of staining was graded on a scale of 0 to 3, with 0 recorded as no staining, 1 as mild intensity, 2 as moderate intensity, and 3 as severe. Only when the positive-staining cell is more than 10 % and its intensity is greater than or equal to 2, it can be considered as true R-cadherin positive cells.

### Statistical analysis

For qRT-PCR analysis, student *t* test was used to assay the differential expression of CDH4 in GC and control groups. For tissue array immunohistochemistry analysis, chi-square test was used to assay the association between R-cadherin expression and clinicopathological variables. Kaplan-Meier analysis and log-rank test were adopted for the plotting of survival curves and for the univariate analysis of relationship between R-cadherin expression and the prognosis of patients with GC. Multivariate Cox regression analysis was used to calculate the relative risk of R-cadherin expression in the overall survival of GC.

## Results

### Comprehensive profiling of membrane proteome in GC cell line BGC-823

**Fig 1:**
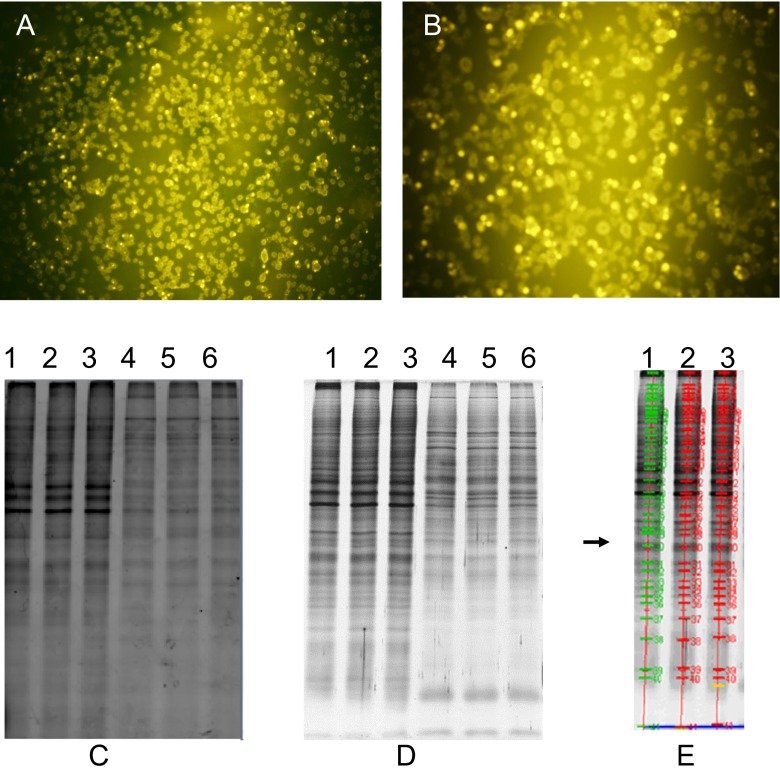
Fluorescence labeling and separation of cell surface proteins in BGC-823 and GES-1 cells. **a**, **b** In situ labeling of cell surface proteins with CyDyes DIGE fluors in BGC-823 (**a**) and GES-1 cells (**b**). **c**–**e** Extracts of fluors-labeled cell surface proteins from BGC-823 (lines 1–3) and GES-1 (lines 4–6) were separated by SDS-PAGE electrophoresis and imaged by typhoon imager scanner using 488-nm laser (**c**) and silverstaining (**d**), and the protein bands cut off for LC-MS analysis were indicated in **e**, *green line*

To explore the cell surface proteins in GC, a novel technology to profile membrane proteome was carried out as described before [26] in two human gastric cell lines (including cancerous BGC-823 and non-cancerous GES-1) to make the results more credible and comparable. Proteins on cell surface of both BGC-823 and GES-1 cells can be specific labeled by fluorescent dye Cy3 and displayed a fluorescent dot or ring distributed in cell surface (Fig. 1a, b). The background was clean, and there were not obvious cell rupture and lysis.

Following membrane protein extraction and SDS-PAGE separation, the gel was scanned on a typhoon imager using 488-nm exciting light to resolve the fluorescent dye-labeled membrane proteins of BGC-823 and GES-1. Results from Fig. 1c showed BGC-823 and GES-1 cells displayed a similar membrane proteome pattern, whereas, a great portion of these proteins were expressed at quite different levels in these two cell lines, indicating the potential roles of these proteins in the regulation of tumor progression. The gel was also stained with silver iron for visualization (Fig. 1d). Then the silver-stained bands corresponding to fluorescent-labeled bands were matched in BGC-823 and GES-1 cells, and the protein bands that are abundant for further LC-MS analysis were cut off from the gel. These bands were indicated in Fig.1e as green lines.

After LC-MS/MS analysis, 118 non-redundant proteins were identified from BGC-823. Gene Ontology database was utilized to analyze the subcellular localization of these proteins. Among them, 89 proteins have their own clear positioning information in Gene Ontology database. These 89 proteins were classified into seven different categories according to their subcellular location, including the plasma membrane or membrane-associated proteins (36/89), nucleus proteins (17/89), cytoplasmic proteins (19/89), secretory proteins (9/89), mitochondrial proteins (4/89), cytoskeleton proteins (3/89), and the endoplasmic reticulum proteins (1/89).

**Fig 2:**
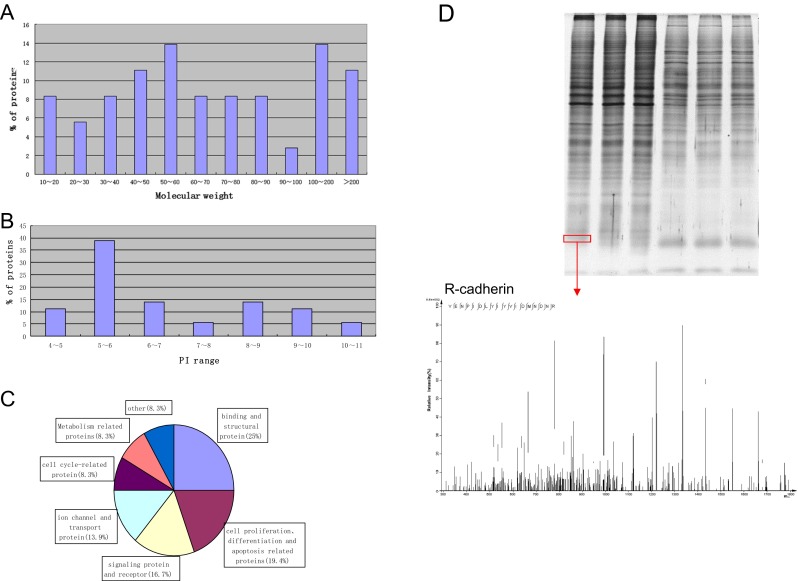
Characteristics of 36 membrane proteins. **a** Molecular weight; **b** PI range; **c** Functional classification; **d** LC-MS of R-cadherin in cell surface of BGC-823 GC cell

These 36 plasma membrane or membrane-associated proteins were further analyzed by bioinformatics tools. Firstly, the ProtParam software (http://www.expasy.org/tools/protparam.html) was used to calculate the GRAVY scores of these 36 proteins. Secondly, TMHMM version 2.0 (http://www.cbs.dtu.dk/services/TMHMM/) was used to calculate the number of TMHs (trans-membrane helical segments, TMHs) of these 36 proteins. Thirdly, the plasma membrane or membrane-associated proteins fall into several categories according to the biology function which gained from gene-ontology (GO) database (http: //www.geneontology.org/) (Fig. 2c). The physical property such as molecular weight (MW) and isoelectric point (PI) of these 36 membrane proteins were also analyzed. The characteristic of these proteins was showed in Table 1. Among all of these 36 proteins, the smallest and the biggest protein molecular weights were 13.2 and 331.8 KDa, respectively. The isoelectric point spreads from 4.65 to 10.64 (Fig. 2a, b). The GRAVY score was between −0.955 and 0.463. There were 14 proteins which have more than one TMD, and the most one named sodium channel protein type 5 subunit alpha is up to 21 (Table 1).

**Table 1:** Characteristics of 36 cell plasma membrane proteins

ID	Protein name	MW	PI	TMDs	GRAVY
Binding and structural proteins					
IPI00796440	Peroxisomal membrane protein 2 (PXMP2)	41447.36	10.64	4	0.165
IPI00382470	Heat shock protein HSP 90-alpha (HSP 86)	98161.31	5.07	0	−0.754
IPI00022426	Protein AMBP	38999.49	5.95	0	−0.341
IPI00290035	Protocadherin-15	216069.72	4.94	1	−0.266
IPI00024034	Cadherin 4 (R-cadherin)	100280.55	4.65	1	−0.301
IPI00218795	L-selectin (SELL)	43617.75	6.96	1	−0.411
IPI00022759	Geranylgeranyl transferase type-1 subunit beta	42396.38	6.37	0	−0.272
IPI00290770	T-complex protein 1 subunit alpha	60462.75	6.1	1	−0.171
IPI00554711	Junction plakoglobin	81744.71	5.75	0	−0.157
Cell proliferation, differentiation and apoptosis related proteins					
IPI00045337	Tumor necrosis factor receptor superfamily member 13C (TNFRSF13C)	18863.8	8.47	2	−0.127
IPI00000690	Apoptosis-inducing factor 1, mitochondrial (AIFM1)	66900.63	9.04	2	−0.227
IPI00013933	Desmoplakin	331774.4	6.44	0	−0.823
IPI00025753	Desmoglein-1	113715.49	4.9	1	−0.285
IPI00179330	Ubiquitin-40S ribosomal protein S27a (RPS27A)	17964.86	9.68	0	−0.489
IPI00017726	3-Hydroxyacyl-CoA dehydrogenase type-2	26923.03	7.65	0	0.227
IPI00220644	Pyruvate kinase isozymes M1/M2	41447.36	10.64	0	−0.132
Signaling proteins and receptor					
IPI00555605	Peripheral plasma membrane protein CASK	104520.22	6.02	0	−0.426
IPI00402234	Ras-associated and pleckstrin homology domains-containing protein 1 (RAPH1)	72873.77	5.91	0	−0.65
IPI00027462	Protein S100-A9 (S100A9)	13241.95	5.71	0	−0.895
IPI00017292	Catenin beta-1 (CTNNB1)	85496.55	5.53	0	−0.175
IPI00010470	Synaptosomal-associated protein 25 (SNAP25)	23315.08	4.66	0	−0.865
IPI00465156	Adenylate cyclase type 4 (ADCY4)	119794.61	7.31	12	0.152
Ion channel and transport protein					
IPI00020542	Solute carrier family 22 member 11 (SLC22A11)	59971.64	8.96	10	0.463
IPI00007188	ADP/ATP translocase 2 (SLC25A5)	32895.2	9.76	3	0.045
IPI00031422	Sodium channel protein type 5 subunit alpha (SCN5A)	224941.21	5.38	21	0.008
IPI00440493	ATP synthase subunit alpha, mitochondrial (ATP5A1)	59750.67	9.16	0	−0.106
IPI00303476	ATP synthase subunit beta, mitochondrial (ATP5B)	56559.96	5.26	0	−0.020
Cell cycle-associated proteins					
IPI00784201	Centrosomal protein of 290 kDa (CEP290)	290544.28	5.75	0	−0.955
IPI00007765	Stress-70 protein, mitochondrial (HSPA9)	73680.41	5.87	0	−0.402
IPI00014238	Lysyl-tRNA synthetase (KARS)	68048.11	5.94	0	−0.454
Metabolism-associated proteins					
IPI00219018	Glyceraldehyde-3-phosphate dehydrogenase (GAPDH)	36053.05	8.57	0	−0.114
IPI00029009	Phosphatidylinositol-4-phosphate 5-kinase type-1 gamma (PIP5K1C)	73260.32	5.17	0	−0.488
IPI00550128	Long-chain-fatty-acid--CoA ligase (ACSBG1)	81258.26	5.73	0	−0.263
Other annotated proteins					
IPI00785084	Ig gamma-1 chain C region (IGHG1)	52286.28	8.84	0	−0.365
IPI00376931	Synaptotagmin-15 (SYT15)	52224.69	8.83	1	−0.258
IPI00292836	Uncharacterized protein C9orf174 (C9orf174)	191100.07	5.74	1	−0.648

To learn more about the gene-related data including genomic, transcriptomic, proteomic, genetic, clinical, and functional information, these 36 membrane proteins were put into GeneCards (http://www.genecards.org/), respectively. According to the information provided by GeneCards database, R-cadherin, a member of the cadherin superfamily which identified from BGC-823, drew our great attention (protein strip and mass spectrum were showed in Fig. 2d). R-cadherin is a calcium-dependent cell-cell adhesion glycoprotein comprised of five extracellular cadherin repeats, a transmembrane region and a highly conserved cytoplasmic tail. Based on studies in chicken and mouse, this cadherin is thought to play an important role during brain segmentation and neuronal outgrowth. In addition, a role in kidney and muscle development is indicated. However, its expression pattern and biological function in GC ignition and progression remain unknown. Therefore, R-cadherin was selected for further research below.

### Low expression of R-cadherin is associated with the tumor differentiation, pTNM stages

**Fig 3:**
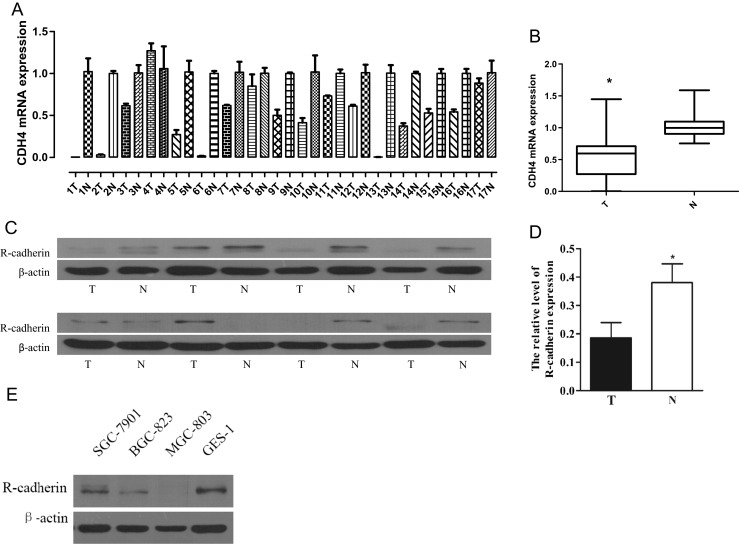
Expression of CDH4/R-cadherin in gastric cancer tissues and cell lines. **a**, **b** qRT-PCR analysis of CDH4 mRNA expression in N vs. T of 17 GC patients. **c**, **d** Western blot analysis of R-cadherin expression in N vs. T of 8 GC patients. T: GC tissues; N: the adjacent non-cancerous mucosa. *P < 0.05, student *t* test. **e** Western blot analysis of R-cadherin expression in immortalized gastric epithelial cell GES-1 and three GC cell lines with different degrees of differentiation and invasive

The expression of R-cadherin was first analyzed in clinic GC tissues and GC cells. Results from qRT-PCR analysis indicated that CDH4 mRNA levels in GC tissues were lower than the paired adjacent non-cancerous mucosa in 88.2 % (15/17) patients (Fig. 3a), and the difference was statistically significant (*P* < 0.05, Fig. 3b).

Results from Western blotting showed the expression of R-cadherin was significantly lower in clinical GC tissues and GC cell lines, as compared to the adjacent normal mucosa to gastric carcinoma and the gastric epithelial cells, respectively (Fig. 3c–e).

**Fig 4:**
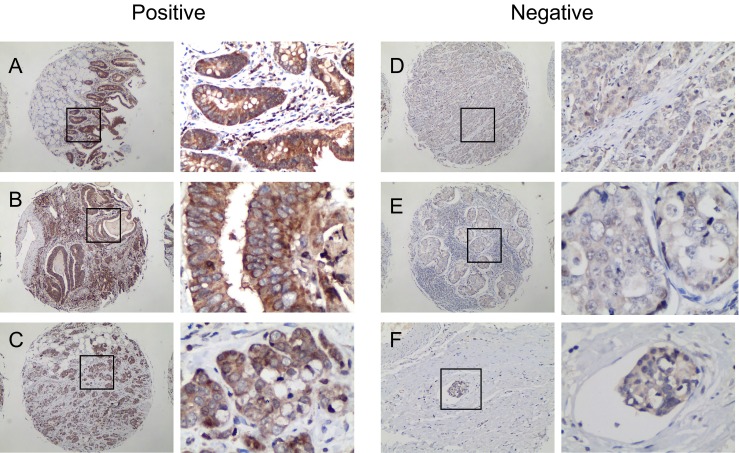
R-cadherin expression in GC and non-cancerous tissues. **a** Mucosa of chronic gastritis; **b** Well-differentiated upper GC; **c** Middle-differentiated upper GC; **d** Poor-differentiated distal GC (pT2N0M0); **e** Poor-differentiated middle GC (pT2N3aM0); **f** Vascular cancer embolus in pool-differentiated GC (pT1bN1M0)

Tissue microarray immunohistochemistry results showed R-cadherin protein expressed dominantly on cell surface of GC and mucosal cells (Fig. 4a–e), similar to the previous report in other tissues (GeneCards database). Its expression deletion in gastric adenocarcinoma (142/169) was higher than that of adjacent non-cancerous mucosa tissues (34/80). The difference between the compared groups was statistically significant (chi-square test, *P* < 0.001).

Clinicopathological analysis revealed the expression of R-cadherin protein was significantly negatively correlated with degree of tumor differentiation, lymph node metastasis, and pTNM stage (chi-square test, *P* < 0.05; Table 2). The patient’s age, gender, and tumor size were not significantly different between the R-cadherin negative and positive expressed groups. Interestingly, R-cadherin expression deletion in upper gastric adenocarcinoma (16/24) was lower than that of middle and distal gastric cancer (126/145) with significance (chi-square test, *P* < 0.05; Table 2). In 16 cases of vascular tumor emboli of gastric cancer, 81 % (13/16) of them were R-cadherin negative expression (Fig. 4f).

**Table 2:** Correlationship between R-cadherin expression and clinicopathological variables

Clinicopathological variables	R-cadherin ICH staining		Chi-square test
	Negative	Positive	*P* value
Gender			
Male	98	19	0.889
Female	44	8	
Age			
≤60	76	11	0.223
>60	66	16	
Tumor site			
Upper	16	8	0.012
Middle and distal	126	19	
Tumor size			
≤5 cm	75	18	0.185
>5 cm	67	9	
Differentiation			
Well and moderate	26	11	0.010
Poor and undifferentiation	116	16	
Invasion depth			
T1-T2	31	10	0.091
T3-T4	111	17	
Lymph node metastasis			
Negative	29	12	0.008
Positive	113	15	
pTNM stage			
I–II	44	14	0.036
III–IV	98	13	

### Low expression of R-cadherin is associated with the poor prognosis in GC

**Fig 5:**
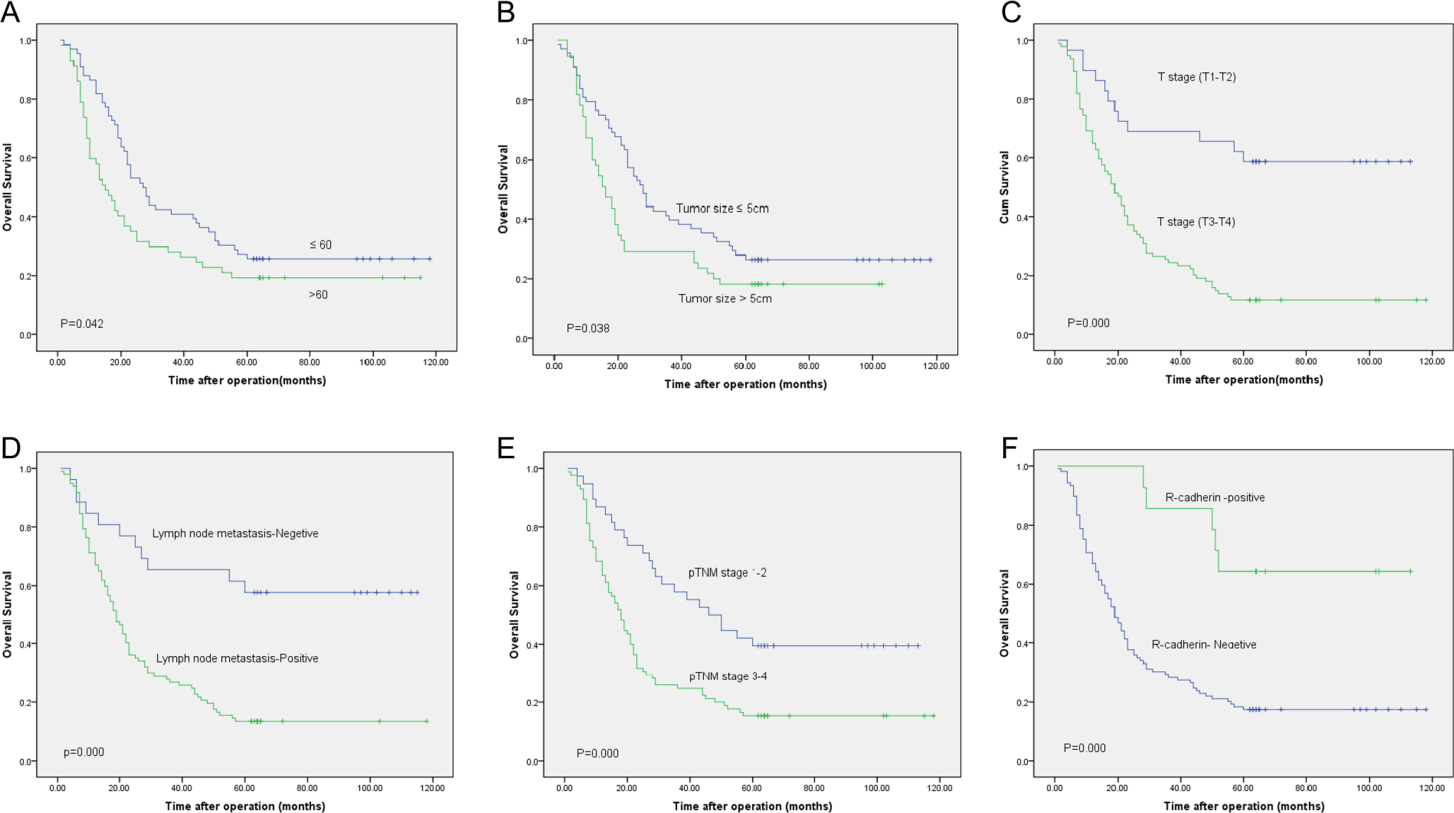
Kaplan-Meier curves and log-rank test for the overall survival of GC patients according to age (**a**), tumor size (**b**), invasion depths (**c**), lymph node metastasis (**d**), pTNM stage (**e**), and ICH staining of R-cadherin (**f**)

Kaplan-Meier analysis showed the patients with R-cadherin positive expression displayed better overall survival than those with negative expression (log-rank test, *P* < 0.01; Fig. 5f and Table 3). Multivariate analysis by Cox regression model revealed that lacking the expression of R-cadherin was a major independent predictor for poor clinical outcome in GC (RR = 5.680, 95 % CI 2.250–14.341, *P* < 0.01; Table 4).

**Table 3:** The overall survival related to several clinicopathological variables with gastric cancer patients

Clinicopathological variables	Patient events		Median survival months	Log-rank test
			(95 % CI, %)	*P* value
Gender				
Male	84	65	21 (15.8–26.2)	0.936
Female	39	30	22 (15.9–28.1)	
Age				
≤60	66	49	27 (20.8–33.2)	0.042
>60	57	46	15 (10.1–19.9)	
Tumor size				
≤5 cm	68	50	28 (22.6–33.4)	0.038
>5 cm	55	45	16 (11.5–20.5)	
Differentiation				
Well and moderate	29	20	23 (17.7–28.3)	0.345
Poor and undifferentiation	94	75	21 (16.7–25.3)	
Invasion depth				
T1-T2	29	12	NA	0.000
T3-T4	94	83	19 (14.7–23.3)	
Lymph node metastasis				
Negative	26	11	NA	0.000
Positive	97	84	19 (15.3–22.7)	
pTNM stage				
I–II	38	23	46 (29.4–62.6)	0.000
III–IV	85	72	18 (14.4–21.6)	
R-cadherin ICH staining				
Negative	109	90	19 (15.6–22.4)	0.000
Positive	14	5	NA	

NA, because the overall survival rates of these patients were more than 50 %, the median survival time cannot be calculated

**Table 4:** Cox proportional hazard regression model analysis of all the patients

Clinicopathological variables	Multivariate analysis		
Age	Relative risk	95 % CI	*P* value
≤60 vs. >60	0.570	0.376–0.864	0.008
Tumor size			
≤5 cm vs. >5 cm	0.911	0.591–1.405	0.673
Invasion depth			
T1-T2 vs. T3-T4	0.340	0.176–0.655	0.001
Lymph node metastasis			
Negative vs. positive	0.495	0.244–1.002	0.051
pTNM stage			
I–II vs. III–IV	0.865	0.500–1.496	0.604
R-cadherin expression			
Negative vs. positive	5.680	2.250–14.341	0.000

## Discussion

Membrane proteomic analysis has been proven to be a promising tool for identifying new and specific biomarkers that can be used for prognosis and monitoring of various cancers [27]. At present, we knew little about the composition and the characteristic of membrane proteins on the cell surface of GC. Membrane proteins have hydrophobic feature; the fraction of membrane proteins in whole cell extractions was quiet low and not easy to be identified without contamination of other intracellular proteins by general proteomic approaches.

Two-dimensional (2D) gel electrophoresis is a powerful technology for protein abundance studies, and it was also the only method available for simultaneous resolution of thousands of proteins. The principle of 2D electrophoresis is based on separation of the proteins according to their charge in the first dimension by isoelectric focusing (IEF) and size in the second dimension by SDS-PAGE. The 2-D DIGE is based on fluorescence prelabeling of protein mixtures before 2D gel electrophoresis. Protein samples are labeled with up to three spectrally distinct charges and mass-matched fluorescent dyes known as CyDye DIGE fluors (cy2/cy3/cy5) and make the quantitative analysis of proteins in a gel. But, unfortunately, it seems not easy to use for the quantitative analysis of membrane proteins because the IEF has always been hampered by the hydrophobic properties.

In order to overcome the deficiency of traditional proteomic method which was used for cell surface proteomics study, a series of methodological studies were performed in recent years. In 2005, a new biotin-avidin chromatography-based membrane proteomic strategy was established by Kazuto Nunomura et al. [28]. In this method, the membrane proteins exposed on intact ES cell surface were selectively labeled with the membrane-impermeable reagent biotin first, then the biotinylated plasma membrane proteins were enriched via affinity capture with immobilized avidin. After that, the biotinylated proteins were separated by 1D SDS-PAGE electrophoresis and identified by LC-MS. Using this method, a series of receptor, transport, and cell adhesion proteins were identified from the undifferentiated mouse embryonic stem cell. In order to further improve the efficiency and purity of membrane protein extraction and enrichment, Sidibe et al. [26] modified the Kazuto Nunomura’s method in 2007. According to Sidibe’s method, the vascular smooth muscle cells (SMCs) were surface-labeled with CyDyes before the labeling of biotin, using the features that fluorescent dyes can specifically tag membrane proteins on the living cultured cell surface. Then, the double-labeled membrane proteins were enriched on avidin column and subsequently separated on large format gradient gels by SDS-PAGE and identified by LC-MS. Compare to the method of Kazuto Nunomura’s, the prominent advantage of this modified method is that CyDye DIGE fluors can specifically target the ε2 site of surface exposed proteins and the fluorescent-labeled cell surface protein can be distinguished from other intracellular contaminants by fluorescence tagging and permits semiquantitative differential expression analysis. In this study, we adopted this modified method to characterize the membrane proteome of GC.

Through this study, a total of 118 separate non-redundant proteins have been identified from GC cell and 89 proteins have the comment information in gene ontology database. Among these 89 identified proteins, 36 were plasma membrane proteins which include 9 adhesion and structural proteins, 7 cell proliferation-, differentiation-, and apoptosis-related proteins, 6 signal and receptor proteins, 5 channel and transporters proteins, 3 cell cycle-related proteins, 3 metabolism-related proteins, and 3 other functional proteins. According to the information of GeneCards database, 20 of these plasma membrane proteins were specifically or dominantly expressed on cell surface. The results of our study further confirmed the reliability and efficiency of CyDye/biotin-labeling approach for cell surface protein study and provided a basic membrane proteome database for biological studies of GC.

Cadherins play a key role in embryogenesis and tissue homoeostasis. The dysregulation of cadherin expression such as E-cadherin has been implicated in tumor progression and metastasis [29, 30]. Apparently, there was a confirmed reports that the loss of CDH1 (E-cadherin) expression in colorectal cancer was associated with infiltrative tumor growth pattern and lymph node metastasis [31].

There were more than 100 superfamily of transmembrane cadherin proteins in human bodies [32]; elucidation of their roles in suppression versus initiation or progression of various tumor types is a young but fascinating field of molecular cancer research [33, 34]. In our study, 2 novel cadherin superfamily membranes, R-cadherin and protocadherin-15, were identified from GC cell. In previous studies, R-cadherin was considered as a key molecular in retinal development [35], but its roles in tumorigenesis was discovered in recent years. In 2004, Elenma Mitto [36] firstly found CDH4, the encoding gene of R-cadherin, was dominant methylated in colorectal cancer and GC. These epigenetic changes can also be detected in the patient’s peripheral blood, suggesting that CDH4 gene may play some special roles in the initiation and progression of neoplasms of digestive system. In 2008, Kucharczak [37] found that the overexpression of R-cadherin in myoblast cell can affect its endogenous N-cadherin and M-cadherin function then inhibit myogenesis and induce myoblast transformation via Rac1 GTPase. In recent years, aberrant promoter methylation of CDH4 was detected in both gastrointestinal tumor and nasopharyngeal carcinoma, respectively [38–40], which demonstrated CDH4 methylation may be a common phenomenon in the process of tumorigenesis. CDH4 was also considered as a potential tumor suppressor gene. In this study, we found R-cadherin protein expression deletion occurred in most of the GC samples, not in adjacent non-cancerous mucosa.

In order to further understand the biological role of R-cadherin in the initial and progression of GC, the relationship between R-cadherin expression and the clinicopathological variables were estimated in 169 GC tissues by immunohistochemical analysis. We found that lacking the expression of R-cadherin was correlated with the grade of cell differentiation, lymph node metastasis, and pTNM stages of GC significantly. In the Western blotting analysis between three GC cell lines with different degrees of differentiation and invasive, we discovered that the expression level of R-cadherin in moderate differentiated gastric cancer cell line SGC-7901 was lower than in immortalized gastric epithelial cell line GES-1. Interestingly, further decreasing expression of R-cadherin occurred in poorly differentiated gastric cancer cell line BGC-823, as well as complete deletion can be seen in gastric mucous adenocarcinoma cell line MGC-803. This founding suggested that the expression level of R-cadherin was associated with differentiation and malignant degrees of GC, which was similar to its trends and characteristics in breast cancer [41], further indicating its role of tumor suppressor gene.

In order to estimate its prognostic value for GC, Kaplan-Meier analysis and log-rank test were adopted to compare the difference of clinical outcome of GC patients with R-cadherin positive and negative expression. Our results indicate that the gastric patients with R-cadherin positive expression have a better 5-year overall survival than those with negative expression. In order to eliminate the interference of other clinical variables in the survival analysis, Cox proportional hazard regression model adjusting age, tumor size, invasion depth, lymph node metastasis, and pTNM stage showed the same trend as the Kaplan-Meier survival curve. R-cadherin expression deletion was considered as a major independent predictor for worse outcome in GC (RR = 5.680, 95 % CI 2.250–14.341, *P* < 0.01).

Protocadherin-15, another member of the cadherin superfamily, is an essential protein in the maintenance of normal retinal and cochlear function. Mutations in this gene will result in hearing loss and Usher syndrome [42, 43]. Currently, a new secreted protocadherin-15 isoform was identified from NK/T cell lymphomas, and it can be used as a potential cell marker [44]. In our study, protocadherin-15 was also identified from BGC-823 GC cell. It could be detected in gastric cell lines using immunochemistry, but its subcellular localization was ambiguous and the results of the Western blotting are not very stable (results were not shown in this paper), which need to be further validated by more excellent antibodies and high-volume clinic GC tissues.

Besides adhesion molecules, many other types of cell surface proteins were also identified from GC cell in our study. Some of them have been reported previously in GC studies [45–47], most of them have not yet been fully explored. The biological role of these proteins remains to be further investigated.

In summary, we have established a fundamental membrane proteomic database of GC. R-cadherin has been identified as a novel tumor differentiation- and progression-related cell surface marker. Its expression deletion in gastric cancer predicts poor clinic outcome.
